# A Review of the Biomedical Applications of Zerumbone and the Techniques for Its Extraction from Ginger Rhizomes

**DOI:** 10.3390/molecules22101645

**Published:** 2017-09-30

**Authors:** Katayoon Kalantari, Mona Moniri, Amin Boroumand Moghaddam, Raha Abdul Rahim, Arbakariya Bin Ariff, Zahra Izadiyan, Rosfarizan Mohamad

**Affiliations:** 1 Department of Mechanical Engineering, Faculty of Engineering, University of Malaya, Kuala Lumpur 50603, Malaysia; 2 Centre of Advanced Materials (CAM), Faculty of Engineering, University of Malaya, Kuala Lumpur 50603, Malaysia; 3 Department of Bioprocess Technology, Faculty of Biotechnology and Biomolecular Sciences, Universiti Putra Malaysia, UPM Serdang, Selangor 43400, Malaysia; Mona_moniri6@yahoo.com (M.M.); Amin.broomandm@yahoo.com (A.B.M.); arbarif@upm.edu.my (A.B.A.); 4 Department of Cell and Molecular Biology, Faculty of Biotechnology and Biomolecular Sciences, Universiti Putra Malaysia, UPM Serdang, Selangor 43400, Malaysia; raha@upm.edu.my; 5 Bioprocessing and Biomanufacturing Research Center, Faculty of Biotechnology and Biomolecular Sciences, Universiti Putra Malaysia, UPM Serdang, Selangor 43400, Malaysia; 6 Malaysia-Japan International Institute of Technology (MJIIT), Universiti Teknologi Malaysia, Kuala Lumpur 54100, Malaysia; Zahra_izadiyan@yahoo.com.my; 7 Institute of Tropical Forestry and Forest Products, Univerciti Putra Malaysia, UPM Serdang, Selangor 43400, Malaysia

**Keywords:** zerumbone, extraction methods, anticancer properties, antioxidant, antimicrobial

## Abstract

Zerumbone (ZER) is a phytochemical isolated from the subtropical Zingiberaceae family and as a natural compound it has different biomedical properties such as antioxidant, anti-inflammatory anti-proliferative activity. ZER also has effects on angiogenesis and acts as an antitumor drug in the treatment of cancer, showing selective toxicity toward various cancer cell lines. Several techniques also have been established for extraction of ZER from the rhizomes of ginger. This review paper is an overview of recent research about different extraction methods and their efficiencies, in vivo and vitro investigations of ZER and also its prominent chemopreventive properties and treatment mechanisms. Most of the studies mentioned in this review paper may be useful use as a knowledge summary to explain ZER extraction and anticancer activities, which will show a way for the development of strategies in the treatment of malignancies using ZER.

## 1. Introduction

**Figure 1:**
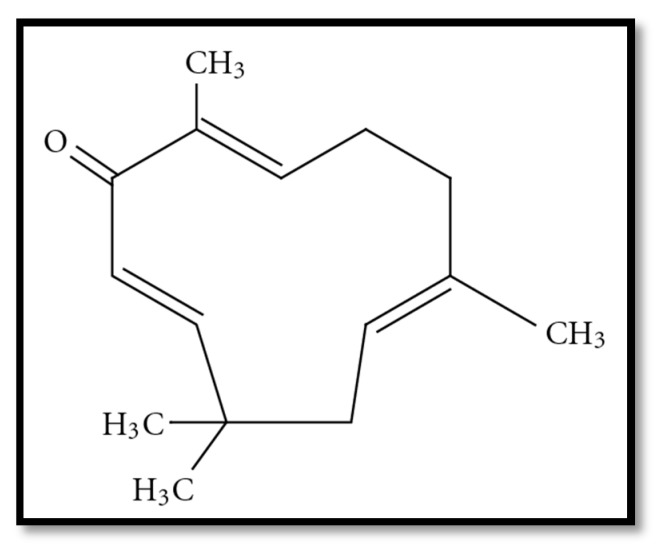
Chemical structure of ZER [28].

Different parts of plants have been used for thousands of years in the human diet. Their leaves, stems and flowers are important to improve the taste, color, odor, as well as the quality of cuisine [1]. Furthermore, scientific research and experiments since the 19th century have shown the extraordinary properties of herb extracts, and their derived condiments and spices as preservatives [2,3,4,5] anti-oxidative [6,7] or antimicrobial agents [8,9] and various other medicinal values. With the increase of illness like cancer, people are now looking for complementary and alternative medicines (CAMs) to treat the disease [10]. Medical vegetables, fruits and spices are included in CAMs due to their contents of active phytochemicals which have many significant health effects [11,12,13,14,15]. It has been determined that due to the presence of drug sources and novel biological compounds a diet rich in seeds, herbs and fruits does not have any side effects and may help in cancer treatment and even prevention [16,17,18,19]. Warm tropical and subtropical areas, are the sources of ginger (the rhizome of *Zingiber officinalis*, Zingiberaceae) [20]. Ginger has attracted attention among anticancer drugs from plants originating in South-East Asia and as one of the oldest herbs. It has been employed by the Chinese people for thousands of years in traditional medicines for headaches, nausea and colds [21,22,23]. In Western and Mediterranean regions, ginger has been used for the treatment of muscular pain and arthritis because it contains anti-inflammatory compounds. It is also useful for treating high cholesterol, ulcers, and migraine headaches [17,24]. Moreover, different parts such as leaves and rhizomes are applied for tea, condiments, spice and other beverages. Rhizomes are used raw or cooked and eaten for flavoring food. Rhizomes are a fibrous and scaly root, with a smooth light brown skin and a pleasant scent. The stems are 60 to 90 cm in height, oblique or erect in shape with smooth leaves [25]. Extensive studies have been devoted to special aspects of ginger’s function. The findings show that South East Asian people have much lower prostate, breast and other cancer type incidence compared to their Western counterparts [26] and it is thought that their diet plays an important role in wellness. ZER, which is obtained from *Zingiber zerumbet* Smith is the major substance in ginger. Rhizomes are the richest part in ZER followed by the leaves [27]. For the first time, in 1956, ZER was obtained from the essential volatile oil extracted from rhizomes of *Zingiber zerumbet*. In 1960, the ZER structure (Figure 1) was investigated and characterized using NMR and X-ray.

ZER (2,6,9,9-tetramethyl-[2*E*,6*E*,10*E*]-cycloundeca-2,6,10-trien-1-one) is a monocyclic sesquiterpene with three double bonds, two conjugated and one isolated, as well as a conjugated carbonyl group, in an 11-membered ring structure. The boiling and melting point of ZER are 321–322 °C at 760 mm Hg and 65.3 °C, respectively [29,30,31]. ZER is also the major substance (59%) in the essential oil extracted from *Zingiber zerumbet* [32] and several evidences refer to ZER’s ability to enhance apoptosis as the main cause for its anti-proliferative activity that has been observed in several tumor cell lines [33]. Several studies on ZER have shown that it is a promising drug for the treatment of different types of cancer such as colon, breast, cervix, and liver cancer and that it inhibits their proliferation and shows selective action towards cancer cells compared to normal ones. They have shown also ZER can prevent cell growth due to some key proteins [34,35,36] and it has an anti-proliferative influence on several cancer cell lines like blood, skin, breast, liver, lung and colon [37,38,39,40] also anti-inflammatory properties [41].

Moreover, using TPSA analysis, the bioavailability of ginger compounds was judged. This descriptor has been reported to correlate with molecular transport which can pass through membranes so allows the prediction of drugs’ transport properties and has been linked to drug bioavailability.

Based on Veber’s rule for good oral bioavailability, the number of rotatable bonds must be ≤10, and the TPSA values ≤140 Å^2^. The rotatable bonds number has been shown to be a very good descriptor of oral bioavailability of drugs. Each single non-ring bond is rotatable bond, bound to a non-terminal heavy (i.e., non-hydrogen) atom. (C–N) amide bonds are not considered due to their high rotational energy barrier. The number of rotatable bonds was found to be appropriate in most ginger compounds. Generally, it has been demonstrated that passively absorbed molecules with a TPSA >140 Å^2^ are thought to have low oral bioavailability. According to the above criteria, the calculated percentages of absorption for ginger compounds ranged between 68.82% and 92.95% [42].

Some research has revealed that no toxic effects are observed on some organs such as kidney and liver after both single and repeated doses of ZER. Jin et al. studied the administration of a dose of 500 mg/kg via intraperitoneal injection to mice as a single dose and different dosages from 5 to 50 mg/kg for repeated doses over a 28 day period. They did a complete evaluation on the water and food consumption, changes in weight and body, histology, as well as serum biochemistry and hematology. In their single and repeated dosage study, they did not observe any significant changes in clinical signs. The obtained results revealed that ZER has toxicity safety in mice cancer treatment [43]. This review proposes to consolidate and present the different ZER extraction techniques as well as recent findings of a wide variety of biomedical applications of ZER in cancer treatment. The usage of ZER as an anti-inflammatory, antimicrobial and anti-oxidant agent also briefly discussed. Anti-gastric ulcer, and immunomodulatory activities of ZER are also presented.

## 2. Different Extraction Methods of ZER

**Figure 2:**
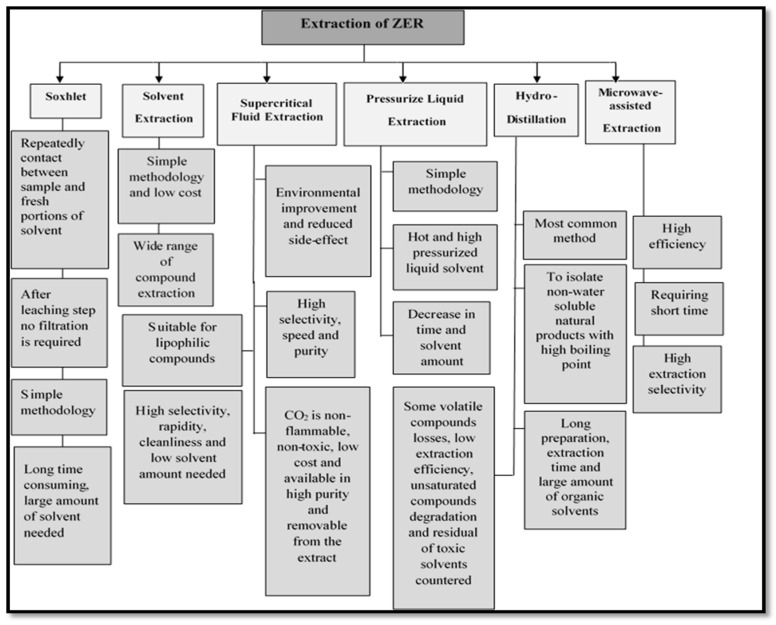
Different methods of ZER extraction.

Several techniques have been used for extract ZER from ginger (Figure 2). Solvent extraction, hydrodistillation (HD), supercritical fluid extraction (SFE), Soxhlet and pressurized liquid extraction (PLE) are the most effective methods which are used. Each method has some advantages and disadvantages. The ideal method should be simple, reliable, faster and economical.

### 2.1. Solvent Extraction

The solvent extraction method has been used widely for the extraction and separation of elements and biocompounds. Water, methanol, ethanol and hexane are the most commonly used solvents in this technique. Ohnishi et al. and Murakami et al. in two different studies, isolated ZER from *Zingiber zerumbet* Smith using methanol at room temperature and concentration under vacuum conditions. The aqueous extract was particioned in (1:1) deionized water and chloroform to give an active CHCl_3_ layer that then was subjected to silica gel chromatography. Finally ZER was obtained by recrystallization from methanol of the EtOAc elute [44,45].

**Figure 3:**
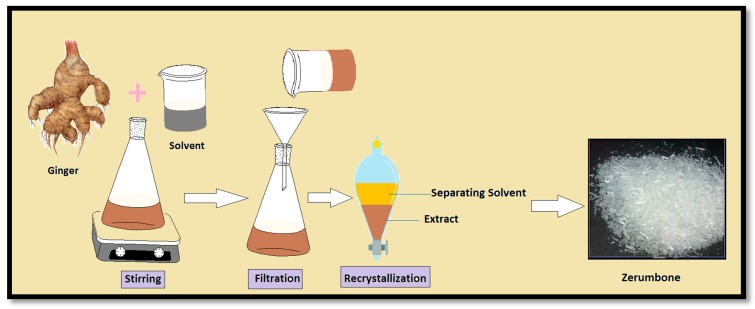
Separation process in solvent extraction.

Recently Tzeng et al. used air-dried *Zingiber zerumbet* for the extraction of ZER. They added 10 L of 95% ethanol to 5 kg of pulverized *Zingiber zerumbet* at room temperature for seven days and with shaking. After evaporation to dryness under reduced pressure, followed by lyophilization about 575 g of dry residue was obtained [46]. Amer et al. and Sriphana et al. also using this method for the extraction of ZER using ethanol at room temperature and analyzed its antimalarial activity and cytotoxicity [47,48]. Numerous studies have been done using solvent extraction for ZER derived from ginger with methanol, ethanol, hexane, etc., in different ratios [46,49,50,51,52,53,54]. The solvent extraction process is shown in Figure 3.

### 2.2. Hydro Distillation

Among the extraction approaches, hydrodistillation (HD), has been the most used extraction method for essential compounds from plant materials such as flowers or wood, and is often applied to isolate non-water-soluble natural products with high boiling points. The HD process involves the complete immersion of the plant species material in water and then boiling. In this method the extracted oils are protected by the surrounding water which is used as a preventive barrier against overheating. The aqueous fraction is obtained by condensing the essential oil vapor. This method has some advantages such as the required materials can be distilled at below 100 °C. In a recent study, Zulazmi et al. isolated ZER from the rhizomes of *Z. zerumbet* Smith. Sliced (0.5–1 mm) rhizomes were placed in a round bottomed flask which was connected to a modified trap and condenser. During the installation process, distilled hexane was added as an organic agent to increase the oil extraction from the condensed vapor. The hexane layer was collected for the next process. By evaporation of the solvent, ZER was obtained. NMR spectroscopy and HPLC were used to identify the ZER and purity (99.8%) confirmation [55]. In another study, Ibrahim et al. isolated ZER using a hydrodistillation method. In order to collect the vaporized steam containing the volatile oil, they placed sliced ginger in a flask containing water and then connected the glassware and heated the mixture. Using cool water, the volatile oil was crystallized. High purity ZER was obtained using hexane [56]. Siavasothy et al. in 2012 isolated some essential oils of the rhizomes and leaves of *Zingiber spectabile* Griff by hydrodistillation. In total they identified 80 compounds in the leaf and rhizome oils [57].

In another study, Das et al. successfully used this method to extract the essential oil from the dried rhizomes of *Zingiber moran*. They identified three major terpenoid fractions namely camphene, citral, and linalool [58]. There have been many studie about using the hydrodistillation method for the ginger oil extraction [37,59,60]. However, conventional techniques such as solvent extraction and hydrodistillation have some disadvantages including long preparation and extraction time and the large amounts of organic solvents needed [53]. Furthermore, some volatile compounds losses, low extraction efficiency, unsaturated compound degradation and toxic solvent residues in the extract may be encountered. Therefore, microwave-assisted, or ultrasound-assisted supercritical fluid and pressurized solvent extraction have been introduced as new techniques.

### 2.3. Supercritical Fluid Extraction (SFE)

The contact between a pressurized solvent and a solid raw material is the basis of the supercritical fluid extraction (SFE) process, which leads to removal of desirable compounds from the solid phase. In the next step, by pressure reduction, the extract is separated from the solvent. This method has been used recently as an alternative to conventional techniques, such as steam distillation and extraction using organic solvents, because the resulting extract is richer in desirable compounds [51,61]. In practice carbon dioxide (CO_2_) is used in most of analytical supercritical fluid extraction (SFE) processes due to several reasons. CO_2_ is non-flammable, non-toxic, relatively low cost and available in high purity and can be removed easily from the extract. Moreover, carbon dioxide has a low critical pressure (74 bar) and temperature (32 °C), respectively. In comparison with liquid pentane, in the supercritical state, CO_2_ is more polar and therefore, is more suitable for extracting lipophilic compounds. The main disadvantage of carbon dioxide concerns the extraction of polar analytes due to its lack of polarity. In the supercritical fluid extraction method, some solvents have been applied for a wide range of applications applications like extraction of metal cations and essential oils, and also the synthesis of polymers and particle nucleation [61].

**Figure 4:**
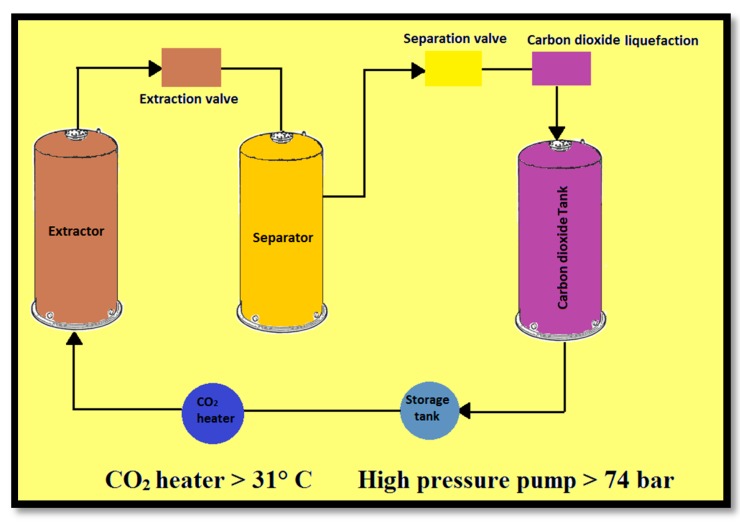
Supercritical fluid extraction using CO_2._

Some reports have been published in the 1990s about the extraction of ginger flavor (i.e., the aromatic and pungent components) from ginger rhizomes using high pressure carbon dioxide. The combination of essential oil and oleoresins in the extract depended on the extraction conditions. The extraction process was done by passing CO_2_ through the sample bed and the content of extract can be controlled by changing the temperature and pressure in the supercritical region of CO_2_ [62] (Figure 4). Norulaini et al. optimized the parameters affecting the supercritical carbon dioxide extraction of non-polar compounds from ginger using Response Surface Methodology (RSM). Dependent parameters for the extraction of ZER were pressure, mass of carbon dioxide and temperature. They found pressure was the most significant variable and at constant temperature, with increasing pressure all of the dependent parameters were enhanced concomitantly. With a combination of temperature and pressure as the two major parameters, the optimized conditions could be determined. By using a Box-Behnken design, extraction at a temperature of 30 °C and a pressure of 55 MPa with a total amount of 30 g of carbon dioxide was used to maximize all the responses [55]. However, the technological conditions required to apply the supercritical fluid extraction method are difficult and expensive which limits its use to the production of special products. Thus, using the supercritical fluid method for insoluble essence extraction from natural products shows outstanding advantages compared to conventional extraction techniques such as steam distillation and Soxhlet extraction. Main features are the high selectivity, speed, cleanliness and the low solvent amount needed.

### 2.4. Soxhlet Extraction

**Figure 5:**
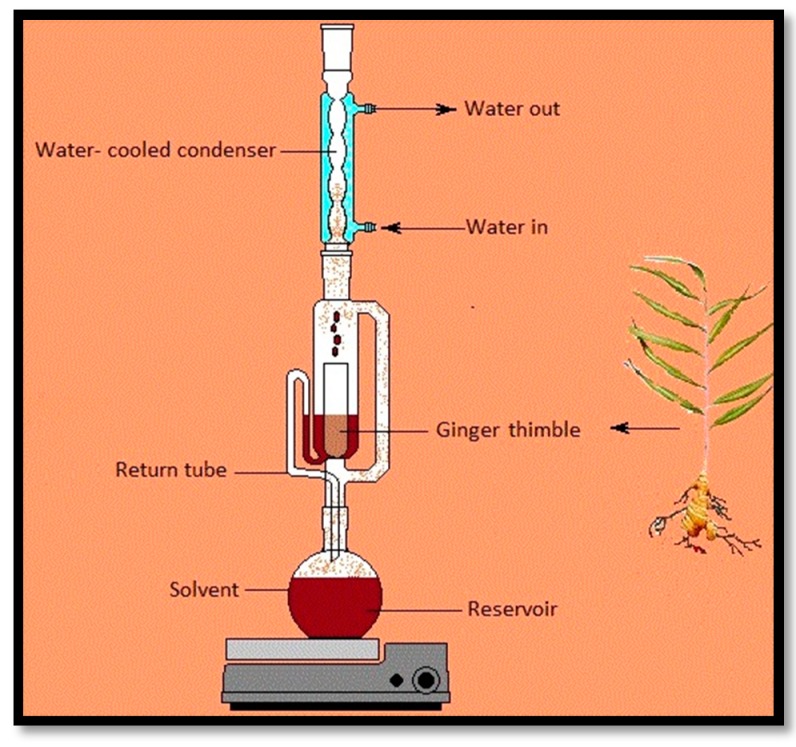
Soxhlet schematic.

Extraction with Soxhlet apparatus is one of the most used pretreatment techniques for solid samples and has been used for more than a century. Generally, Soxhlet extraction is a hybrid continuous–discontinuous method and can be used as a batch system (Figure 5). This method has some advantages such as the repeated contact between the sample and fresh portions of the solvent. After the leaching step no filtration is required. Sample throughput can be improved by simultaneous extraction in parallel, and moreover the basic equipment is inexpensive. This is a non-matrix dependent method which needs simple training due to the simple methodology [63]. However, the most important disadvantages of this method are the long time consumption, and the use of a large amount of solvent which leads to environmental problems. In some cases, extraction of samples at the solvent boiling point can cause the thermal decomposition of the target compounds.

Hamdi et al. used different solvents-Soxhlet extracts from *Curcuma zedoaria* rhizomes for testing on two human cancer cell lines. Briefly they soaked 1.0 kg of *Curcuma zedoaria* powder in hexane for three days. Then, using 5 L of hexane, the solvent-containing extract was filtered. In the next steps, they applied a rotary evaporator, CH_2_Cl_2_ and EtOAc. The insoluble EtOAc extract was further extracted with methanol and then subjected to Soxhlet extraction. They found the hexane extract was able to improve apoptosis in MCF-7 cells by inhibiting the proliferation of the cancer cells [64]. The powder of *Zingiber zerumbet* was extracted using the Soxhlet method by Shieh et al. The fresh rhizome was subjected to reflux with ethanol two times for six hours and at 80 °C. Then the extract was subjected to dilution with water and apportioned with (C_2_H_5_)_2_O. Their results showed that the carbohydrate substrate type, light regime, incubation temperature and agitation speed, have an influence on the production of ZER [54] these look like conditions for a fermentation—explain what they are doing here. This method has been used in many studies as an extraction technique of ZER [30,37,63,65,66] due to its very simple methodology and inexpensive equipment.

### 2.5. Pressurized Liquid Extraction (PLE)

Extraction using pressurized liquids has attracted attention as a good technique for organic solvent extraction. Elevated temperatures and pressures are essential factors in this method which uses liquid solvents to prepare samples. PLE has a simple methodology and needs hot and high pressure liquid solvents which leads to time and solvent savings. Norfazlina et al. used a PLE method to extract ZER from *Zingiber zerumbet* and *Nigella sativa*. They used rhizomes of *Z. zerumbet* chopped into small pieces, then dried and ground into a fine powder. Then an Accelerated Solvent Extraction (ASE) machine was used as extraction equipment. The extraction time was 10 min for one cycle using 1500 psi at 80 °C for 24 h. They investigated the interactive effects of the extract on the HL60 cell line at combination doses in non-constant ratio. As a conclusion the combination between *Zingiber zerumbet* and *Nigella sativa* supplied the antagonist interactive effects and showed that there is no combination that provides a safe drug [67].

### 2.6. Microwave-Assisted Extraction

Recently, this extraction technique has been found to be an efficient method. Microwave-assisted extraction has high efficiency when fresh solvents are continuously in contact with the extraction vessel [68]. Ghasemzadeh et al. [69], investigated several variables such as ethanol concentration, microwave power, time of irradiation and liquid/solid ratio affecting the microwave extraction of ZER from its plant sources. They added different solvents (chloroform, methanol, ethanol and *n*-hexane) to ZER powder and then applied microwave extraction to the resultant mixture. After cooling the solution and vacuum filtration, they freeze dried the residue. Based on their findings, a 38 mL/g liquid/solid ratio, 38.5 s irradiation time, 518 W of microwave power and ethanol concentration of 44% were the best conditions.

## 3. Biomedical Applications of ZER

### 3.1. Anticancer Properties of ZER

Nowadays in the world, cancer is one of the most deadly illnesses. In Western medicine, diverse chemotherapeutic, cytotoxic and immune-modulating agents are used for cancer treatment. Besides the high cost of treatment, these drugs have critical side effects, so studies seeking for alternative treatments that minimize both costs and side effects continue [70]. ZER has several pharmacological properties, and different anticancer, anti-oxidant, anti-inflammatory and antibacterial activities have been reported [71]. Several studies have demonstrated that ZER has both in vitro (Table 1) and in vivo (Table 2) anti-cancer properties at different dosages and concentrations [72]. ZER yields cytotoxic or protective effects in different settings and has anti-proliferative properties towards several cancer cell line such as breast (MCF-7), liver (HepG2), kidney (A293), lung (H1299), colon (COLO205, LS174T, L8174, LS189 and COLO320DM), brain (GBM8401) and blood (CEMss, WEHI-3B and KBM-5) with minimum effects on normal cells [73,74]. The cytotoxic influences of ZER against cancer cells are due to the α,β-unsaturated carbonyl group in its structure which shows significant interactions with biological molecules. Briefly, this group removes intracellular glutathione (GSH) effectively by forming a Michael adduct with it, so the intracellular redox potential (E) increases, which stops the proliferation of cancer cells. On the other hand, in normal cells, a differences in average intracellular redox potential happens whereby it is higher than in cancer cells, so the ZER is harmless to normal cells [75,76].

#### 3.1.1. Blood Cancer (Leukemia)

**Figure 6:**
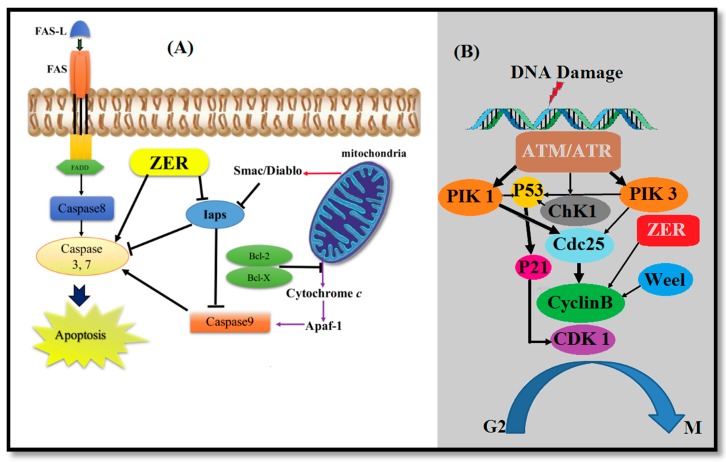
Proposed signaling pathways of ZER-induced apoptosis in cancerous cells (**A**); The cytotoxicity effects of ZER on leukemia cells through cell cycle arrest (**B**).

Human chronic myelogenous leukemia (CML) has a defined genetic alteration association that makes it different from most other types of cancers and is known as a myeloproliferative malignancy [92]. As shown in Figure 6A, ZER has cytotoxic effects on leukemia cells through cell cycle arrest and Fas- and mitochondria-mediated apoptosis [107].

Several studies have revealed that the generation of TPA-induced superoxide anion is effectively suppressed by ZER. This anion is generated from two main sources: xanthine oxidase in AS52 Chinese hamster ovary cells and NADPH oxidase in dimethyl sulfoxide-differentiated HL-60 human acute promyelocytic leukemia cells [28]. The antiproliferative activities of ZC-B11 on HepG2, MCF-7, CEMss, and MDAMB-231 cell lines showed IC_50_ values of less than 30 μg/mL. However, the lowest IC_50_ value of ZC-B11 on CEMss suggested preliminarily that ZC-B11 has high anticancer properties against T-acute lymphoblastic leukemia without showing any cytotoxic effect on human blood mononuclear cells up to a concentration of 50 μg/mL [108]. More studies found that ZER can inhibit HL-60 cell growth, in a time and concentration dependent pathway. Reductions in cyclin B1/CDK1 protein level and induction of G2/M arrest have been achieved after treatment with ZER in HL-60 cell analysis. Moreover, as shown in Figure 6B, ZER has inhibition properties against the proliferation of the NB4 cell line with acute promyelocytic leukemia cells source, by the induction of G2/M phase cell cycle arrest associated with a decline of cyclinB1 protein and phosphorylation of ATM/Chk1. Evidences showed that on leukemic cells, ZER has cytotoxic effects on human myeloid (KBM-5) [36] and human acute lymphoblastic leukemia (Jurkat) cell lines [109].

#### 3.1.2. Breast Cancer

Breast cancer is the second most common illness among women. Several studies have found inhibition activities of ZER on key proteins for prevention of cancer cell growth. Based on the results of Fatima et al. ZER had significant binding with the tumor necrosis factor, kinase κB (IKKβ) and the nuclear factor κB (NF-κB) component proteins along the TNF pathway. They suggested that ZER can exert its apoptotic activities by inhibiting the cytoplasmic proteins.

In 2015 Fatima et al. found that the apoptotic activities of ZER can be used for cytoplasmic protein inhibition. IKKβ kinase activated the NF-κB. ZER could inhibit this activation and also has binding ability to the NF-κB complex in the TNF pathway. When both proteins were blocked, inhibition of cell proliferating proteins occurs [87]. Moreover, Sehrawat et al. demonstrated that ZER inhibits in vivo (MDA-MB-231 cells) and in vitro (MCF-7 and MDA-MB-231 cells) human breast cancer cells growth in association with apoptosis induction. They used western blotting to find changes in protein expression and for knockdown of Notch2 or Presenilin-1 protein, small hairpin RNA or small interfering RNA Transfection were applied. Apoptosis and migration of cells were analyzed by flow cytometry and a chamber assay, respectively. Using of ZER leaded to cleavage increasing of Notch2 in SUM159, MCF-7 and MDA-MB-231 cell line. Furthermore, following treatment by ZER, the levels of cleaved Notch4 and Notch1 proteins were decreased. Higher cleavage of Notch2 in cells exposure with ZER take place by Notch transcriptional activation and induction of Presenilin-1 protein. Using of ZER leads to inhibition of cell migration and induction in apoptosis completed by knockdown of Notch2 protein. Briefly, their research showed that ZER is able to activate the Notch2 which proved its proapoptotic and anti-migratory properties [86].

#### 3.1.3. Liver Cancer

Cancer of the liver is the third most common cancer worldwide [110]. This kind of cancer is closely linked to chronic hepatitis B infection and can commonly kills almost all affected patients within a year [76].

**Figure 7:**
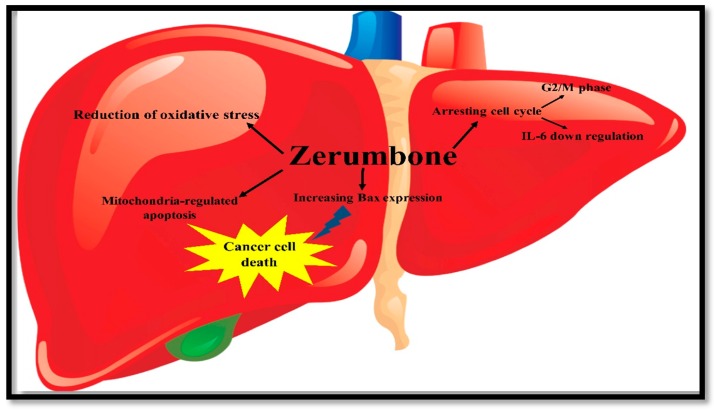
Different approaches of ZER can effect on liver cancer cells.

Briefly, in 2010 Taha et al. assessed the antitumourigenic effects of ZER in rats which were exposed to injection of dietary 2-acetylaminofluorene and diethylnitrosamine. Then rats also received ZER using intraperitoneal injection in different dosages for 22 times during 11 weeks. They also used histopathological evaluations. The results showed the protective role of ZER against the carcinogenic effects of dietary 2-acetylaminofluorene and diethylnitrosamine. Treatment with ZER leads to significant lower amount of serum alanine transaminase, aspartate transaminase, α-fetoprotein and alkaline phosphatase compared with rats with untreated liver cancer. There was a significant increase in liver malondialdehyde concentrations in untreated rats which indicates peroxidation of hepatic lipids. Moreover, the hepatic tissue glutathione concentrations decreased significantly. In the livers of dietary 2-acetylaminofluorene and diethylnitrosamine rats, ZER treatment leads to an increase and decrease in Bax and BCL-2 protein expression, respectively, that suggested increased apoptosis. As revealed in Figure 7, induction of mitochondria-regulated apoptosis of liver cancers are the key pathways of ZER effects [105].

In another study Sakinah et al. found that ZER can induce apoptosis in liver cancer cells due to antiproliferative activity upon HepG2 cells in a time-course manner. Furthermore ZER was able to slow the non-malignant Chang liver and MDBK cell line proliferation. Using ZER leads to an apoptosis process involving pro-apoptotic Bax protein upregulation and the suppression of anti-apoptotic Bcl-2 protein expression. Some changes took place in the level of Bax-Bcl-2 antagonistic proteins. These changes were independent of p53 since ZER did not have any influence on the p53 levels. They used western blotting analysis for immunoassay to qualitatively confirm Bax protein that revealed the Bax protein distribution in cells treated with ZER [105].

#### 3.1.4. Colon Cancer

Recent studies on different human colonic adenocarcinomas have demonstrated that ZER had suppressive influence against inflammatory and oxidative stress factors such as generation of free radicals, nitric oxide synthase expression and tumor necrosis factor-α release [111]. Previously, Yodkeeree et al. showed in their research, the inhibition effects on proliferation of human colonic adenocarcinoma in a dose-dependent-manner (LS174T, LS180, COLO205, and COLO320DM) cell line, while less influence was observed on the normal human colon fibroblasts (CCD-18Co) growth and (2F0-C25) normal human dermal cells. They found that ZER shows TRAIL-induced apoptosis ability in HCT116 colon cancer cells which is in correlation with the upregulation of TRAIL death receptor (DR) 4 and DR5 through the reactive O_2_ species-mediated activation of extra-cellular signal-regulated kinase ½ and p38 mitogen-activated protein [112]. Furthermore, according to Murakami et al.’s reports, ZER can significantly induce in a time and concentration-dependent manner, the expressions of TNF-α, IL-1α, IL-1β and IL-6 in human colon adenocarcinoma (Caco-2, Colo320DM, and HT-29) cell lines [21].

#### 3.1.5. Lung Cancer

Lung cancer is the main cause of death around the world. Non-small cell lung cancer (NSCLC) is the most common type of lung cancer [113]. Advanced treatments, like surgery and chemotherapy offer limited improvement in the survival of patients with NSCLC [82]. Recently, Hseu et al. tested the anti-EMT and anti-metastatic properties of ZER in TGF-β1-stimulated human lung cancer (A549) cells as a NSCLC. ZER inhibits the EMT induction of TGF-β-via upregulation of E-cadherin and down-regulation of Smad2 signalling pathways. Moreover, E-cadherin downregulation and EMT-linked signaling regulators changes, showing the high sensitivity of A549 cells to TGF-β1 which could propagate the EMT process in cancer cells. They also found, that exposure to ZER leads to TGF-β1-induced increased cancer cell migration, invasion and formation of colonies [82]. Hu et al. investigated the anticancer activity of ZER on NSCLC cells and explored the p53 signaling which has an important role in cell death. Their findings showed that ZER has the ability to induce mitochondrial apoptosis and enhances the susceptibility to cisplatin in NSCLC cells, which acts as activation mediated through p53 signaling and promotion of ROS generation [84].

#### 3.1.6. Pancreatic Cancer

During the past years, pancreatic carcinoma has become a common cancer around the world. Zhang et al. reported the effect of ZER on pancreatic cancer cells. According to their results, in a concentration and time dependent manner, ZER caused inhibition of the viability of PANC-1 cells and ZER induced apoptosis of these cells. Moreover, in ZER-treated PANC-1 cells upregulation of p53 protein and high level of p21 were detected. They also found ZER had the same antitumor properties on SW1990 and AsPC-1 pancreatic carcinoma cell lines [79].

#### 3.1.7. Gastric Cancer

Gastric cancer is known as the fourth most frequently diagnosed cancer and the second main cause of cancer-related mortality. Tsuboi et al. showed in 2013 that ZER has reduction and inhibition effects on VEGF production and NF-κB activity and tumor angiogenesis in human gastric adenocarcinoma (AGS) cells, respectively. They studied the vascular endothelial growth factor (VEGF) in the basal state and ZER-treated gastric cancer cell lines by real time RT-PCR and enzyme-linked immunosorbent assay (ELISA). They also investigated the changes in proliferation of gastric cancer cells by a WST-1 assay. Briefly, among the six tested gastric cancer cell lines, GS cells had the highest expression of VEGF. In AGS cells, ZER inhibited the proliferation of cells, expression of VEGF and NF-κb activity. The reduction in NF-κB activity and VEGF expression took place in AGS cells by ZER-treated cells [114,115].

### 3.2. Anti-Inflammatory Activity

ZER has demonstrated anti-inflammatory activities [116]. In 2009, Ganabadi et al. choose rats with collagen-induced osteoarthritis to evaluate the ZER effects on major histocompatibility complex type II (MHC II) expression in synovial membranes. They compared these cells with a corn oil and ZER-treated group. Their results revealed that in the ZER-treated group, the level of MHC class II-immunoreactivity was significantly reduced compared with the corn oil one and only type A cells were positive for MHC II staining. Briefly, according their findings, ZER has inhibition activities against the antigen presenting cells (type A) and reducing the inflammatory process in osteoarthritis [117]. Somchit et al. also used ZER for its analgesic and anti-inflammatory properties in rats. The results showed that ZER effectively inhibits induction in inflammation by both prostaglandin E2 and λ-carrageenan, which was statistically similar to the nonsteroidal anti-inflammatory drug (NSAID) piroxicam. They also found that ZER can act as pain inhibitor in the sabdorminal writhing test similar to the NSAID [88].

Chien et al. explored the antinociceptive and anti-inflammatory effects of ZER on arthritis. Their findings revealed that ZER has the ability to inhibit inducible nitric oxide synthase and cyclooxygenase (COX)-2 expressions as well as production of NO and prostaglandin E2 (PGE2), moreover ZER induced heme oxygenase (HO)-1 expression with dose-dependent pattern in lipopolysaccharide (LPS)-stimulated RAW 264.7 cells [118].

In a recent study, the immunomodulatory influences of ZER on in vitro antigen-presenting dendritic cells (DCs) and its potential therapeutic effect on ovalbumin (OVA)-induced T helper 2 (Th2)-mediated asthma in mice were investigated.

By using the ZER, polarization an enhancement is observed in the polarization and proliferation of T cell through lipopolysaccharide-activated bone marrow-derived DCs in an allogeneic mixed lymphocyte reaction. The results showed that ZER has an antiallergic effect on viamodulation of Th1/Th2 cytokines in an asthmatic mouse model [54].

Wenhong et al. reported the pretreatment effects of ZER on a rat acute necrotizing pancreatitis model induced by (C_26_H_45_NO_7_S). The I-κBα, NF-κB protein ICAM-1 and IL-1β mRNA expression were investigated. Their results demonstrated that the levels of ALT, AMY, sPLA2 and AST and hepatic and pancreatic tissues histopathological assay were markedly reduced due to the use of ZER. ZER also inhibits NF-κB protein and downregulates ICAM-1 and IL-1β mRNA [119]. Moreover, Al-saffar et al. in 2011 used *Channa striatus* extract and ZER in rat synovial membrane, for comparison of the immunoreactivity of some osteoarthritis-related neuropeptides against monosodium iodoacetate which shows induction effects on osteoarthritis changes of the knee. They used a serum containing PGE2 and PGF2α for evaluation of their role during osteoarthritis events and post oral treatment application. Their results showed lower pathology scores accompanied with markedly improved immunoreactivity in ZER-treated groups compared to other groups [120]. Dietary ZER also can prevent ultraviolet B-induced cataractogenesis in mice. Chen et al. in one of their studies, exposed 6-week-old female mice fed with different dosages of ZER to UVB. Then, cataract examination was done on all mice along with lens opacity scoring, in correlation with malondialdehyde levels, glutathione, GSH reductase, glutathione peroxidase, and superoxide dismutase in the lens. They found that 100 mg/kg dietary ZER after exposure of UVB leads to decreased lens opacity scores [106]. Furthermore, in another study, they found that ZER act as a strong NF-κB inhibitor inflammation modulator on UVB-induced corneal damages in a mouse model. UVB irradiations caused seriously damage to cornea, epithelial exfoliation, sustained inflammation, apparent corneal ulcer, and infiltration of polymorphonuclear leukocytes. According their results, dietary ZER inhibits against NF-κB, iNOS, and TNF-α with concomitant reduction of MDA accumulation and increase of GSH and GR levels in the mouse [121].

### 3.3. Antioxidant Activity

ZER has antioxidant activity through two main pathway, which include reactive oxygen (RO) attenuation and N_2_ species generation. Hamdi et al. used NG108-15 neuroblastoma-rat glioma hybridoma cells for investigation of the ZER-epoxide effect on H_2_O_2_-induced oxidative stress. In assessment of oxygen radical antioxidant capacity (ORAC), a strong antioxidant activity was observed [122]. In a recent study, the gastroprotective effect of ZER was evaluated on an ethanol-induced gastric ulcer model in rats. First, the authors did a pre-treatment by ZER on rats and then exposed them to acute gastric ulcers induced by absolute ethanol administration. The findings showed that ZER can promote ulcer protection that might be related to mucus integrity maintenance, antioxidant activity, and HSP-70 induction. Moreover, gastric mucosa was protected by intragastric administration of ZER from the aggressive effect of ethanol-induced gastric ulcer, in the same time with reduced submucosal edema and leukocyte infiltration [123].

Mesomo et al. reported the antioxidant activity of ZER using the phosphomolybdenum reduction method. They found the most significant antioxidant activity was (931.67 ± 2.51 mg of α-tocopherol/g of extract) [124].

Wan-Shin et al. reported that ZER induced activation of NF-E2-related factor 2 (Nrf2) and expression of heme oxygenase-1 (HO-1) in hairless mice (dorsal skin).They compared the levels of HO-1 protein in the skin of ZER-treated Nrf2 wild-type and Nrf2 knockout mice, and Nrf2-deficient mice expressed HO-1 protein to a much lesser extent than the wild-type animals following application of ZER. Treatment of mouse epidermal JB6 cells with ZER caused a marked increase of Nrf2 nuclear translocation followed by the promoter activity of HO-1, and also direct binding was enhanced the Nrf2 to the antioxidant response element [104].

Based on Wan-shin et al.’s findings treatment with ZER led to nuclear translocation enhancement of nuclear factor E2-related factor 2 (Nrf2), a transcription factor regulating expression of phase 2 detoxifying/antioxidant enzymes including HO-1. Moreover, ZER application onto the dorsal skin of HR-1 hairless mice also induced HO-1 protein expression in a time dependent manner, with maximal induction observed at 6 h [125].

### 3.4. Immunomodulatory Activity

Keong et al. evaluated the effect of ZER on lymphocyte proliferation in different induction cells, such as mice splenocytes and thymocytes, human peripheral blood mononuclear cells, PBMC), cell cycle progression and cytokine (interleukin 2 and 12). According their findings, the use of ZER leads to activated mice splenocytes, thymocytes and PBMC in an amount dependent assay with 7.5 μg/mL as the optimum concentration. A prominently upregulation at 24 h and decrease from 48 h to 72 h were observed in the production of human interleukin-2 and human interleukin-12 cytokines in culture supernatant of ZER-activated lymphocytes [96]. The progression and induction of cytokine (IL-2 and IL-12) are affected by ZER. This was demonstrated by the proliferation of ICFmice thymocytes and splenocytes and human peripheral blood mononuclear cells (PBMC). Entrance of high population of PBMC to G2/M phase is caused by ZER treatment [96].

Sriphana et al. investigated the in vitro antimalarial activity of ZER against *Plasmodium falciparum*. Based on their findings the carbonyl group played a significant role in the cytotoxicity [48].

According to Keong et al.’s findings, ZER has effect on the proliferation, cell cycle progression, and induction of cytokine (IL-2 and IL-12) of immune cells in vitro. This was demonstrated by the proliferation of ICF mice thymocytes and splenocytes and human peripheral blood mononuclear cells (PBMC) [96].

### 3.5. Anti Gastric Ulcer Activity

In rats, ZER has gastroprotective effects against an ethanol-induced gastric ulcer model. According to the findings of Sidahmed et al., ethanol-induced gastric ulcers have an aggressive effect on gastric mucosa and ZER can protect them by reduced submucosal edema and leukocyte infiltration. Their results also showed that ZER promoted the protection of ulcers, which might be attributed to the mucus integrity maintenance and induction of HSP-70 [123].

In another study, the gastroprotective activity of ethanol extract of *Z. simaoense* rhizome was investigated in rats. Baiubon et al. found that *Z. simaoense* extract strongly inhibited the formation of gastric ulcers in all gastric ulcer models [126].

Chantharangsikul et al. presented in their study the gastroprotective activity of 95% ZER. Based on the reported results, ZER highly increased the visible gastric mucus secretion and showed a tendency to increase the secretory rate of soluble gastric mucus [127].

### 3.6. Antimicrobial Activity

*H. pylori* is the most prevalent bacterial infection that affects approximately half the world population. ZER also exhibits significant antibacterial action against this bacterium. *H. pylori* has a spiral shape and is a Gram-negative pathogen which colonizes the gastric mucus layer and adheres to the epithelium [128].

In another study, Tzeng et al. investigated the effect of ZER on hepatic lipid metabolism in Syrian golden hamsters which were on a high-fat diet (HFD) and then dosed orally with ZER at different dosages. The plasma levels of total triglycerides and cholesterol in hepatic tissue, and also insulin resistance in a homeostasis model assessment were lowered, especially in the ZER-treated group. Briefly, ZER can improve insulin sensitivity, decrease lipogenesis, and increase lipid oxidation in the liver of HFD-fed hamsters, pointing to a potential application in the treatment of non-alcoholic fatty liver diseases [103]. In 2013, Kumar et al. synthesized two new compounds—azazerumbone 1 and azazerumbone 2—using a ZnCl_2_-catalysed Beckmann rearrangement of ZER oxime. These two compounds have antibacterial activities against four different food-borne pathogenic bacteria which were analyzed and then compared with ZER. They used pour plate method against different Gram positive and negative bacteria and found that azazerumbone 2 had better activity than ZER. Also, these compounds showed prominent protection against sodium azide-induced mutagenicity of Salmonella typhimurium strains TA 98 and TA 1531. Moreover, azazerumbone 2 had better antimutagenic and antibacterial activities compared with azazerumbone 1 [129].

Rana et al. reported a significant antifungal activity against some phytopathogenic fungi, such as *Sclerotium rolfsii*, *Rhizoctonia solani* and *Macrophomina phaseolina* compared with hexaconazole. The value of EC_50_ was 59.3, 39.6 and 147. 4 respectively which are higher than the hexaconazole values [130].

## 4. Conclusions

Natural compounds are small molecules from biological sources, such as animals, microorganisms, plants and marine organisms [131]. Herbs and spices are considered safe and effective against certain deceases. These useful compounds have been used in many disease treatments. Actually, more than 60% of approved anti-tumorigenic drugs derive from natural sources [132]. *Zingiber zerumbet* Smith is a plant which grows mainly in Southeast Asia [131]. As a main component extracted from the essential volatile oil of *Zingiber zerumbet* Smith, ZER is a phytochemical compound used for its properties against virus infection, inflammatory diseases, treatment of leukemia and cancers [52]. ZER in total has three double bonds (one isolated and two conjugated), as well as an 11-membrane ring structure containing a double conjugated carbonyl group [21].

In this review paper, different extraction techniques such as solvent extraction, hydro- distillation, supercritical fluid, Soxhlet and pressurized liquid extraction were summarized.

Moreover, many studies have clearly showed that ZER has both in vitro and in vivo biological properties at different dosages and concentrations. ZER possesses significant anticancer properties towards different cancer cell lines and low effects on normal cells. ZER inhibits the growth of cancer cells through apoptosis induction and cell cycle arrest [34]. Herein, the detailed mechanism of cancer cell death in multiple organs was clarified. Furthermore, several studies have investigated the anti-inflammatory influences on the proliferative and exudative phases of inflammation and justified the the wide use of ZER for the treatment of inflammatory conditions. Based on these studies, there is a strong link between promotion of tumor, oxidative stress and inflammation, and ZER also acted as an anti-carcinogenic agent [21]. The antioxidant ability of ZER due to reactive oxygen attenuation, so it could occur that it has anti-cancer-related inflammation potential activity which has been investigated may be mediated through ZER antioxidant activity.

The results from all the studies and research mentioned in this review paper are definitive evidences that ZER is a powerful compound in the treatment of cancer and several other diseases and it possesses different beneficial in vitro and in vivo biological activities. It is nevertheless essential to do more animal studies and human clinical trials to determine the efficacy, safety and usefulness of ZER as an intended pharmaceutical drug.

## Figures and Tables

**Table 1:** In vitro biological effects of ZER.

Organ	Cell Line	Biological Effects of ZER	References
Pancreatic	INS-1 rat pancreatic b cells	Enhances the viability of INS-1 b cells (high glucose). ZER can attenuate significantly the apoptosis of high glucose-induced INS-1 cells.	[77]
	Human pancreatic carcinoma (PaCa)	Excellent inhibitor of Jak2/Stat3, which inhibits the growing of promigratory gene as well as the expression and migration of cancer cells.	[78]
	Human pancreatic carcinoma (PANC-1 and SW1990)	Effect on cell viability inhibition and induces apoptosis in time-dependent manner.	[79]
	Human pancreatic carcinoma (PaCa)	By inhibition of NF-1D705B and NF-κB-dependent proangiogenic gene products, ZER can inhibit PaCa-associated angiogenesis.	[80]
Lung	ATCC-HTB-57 cells	Some novel compounds fabricated through azazerumbone conjugation with 2,4-dihydroxychalcones. These compounds showed anti-proliferative activity against the LU-1, Hep-G2, MCF-7 and SW480 human cancer cell lines.	[81]
	TGF-β1-stimulated human (A549) cells	Demonstrates the anti-EMT and anti-metastatic properties of zerumbone in A549 lung cancer cells under TGF-β1-stimulation.	[82]
	Human small cell lung carcinoma (NCI-H187)	Inhibits the HSP 27 protein as a monomeric form of. Derivative of ZER induces strong cytotoxicity.	[83]
	Human non-small cell lung cancer (NSCLC) cells	The viability of NSCLC cells, significantly impaired by treatments of in a dose-dependent manner and NSCLC.	[84]
Liver	ATCC-HTB-22 cells	Some novel compounds fabricated through azazerumbone conjugation with 2,4-dihydroxychalcones. These compounds showed anti-proliferative activity against the LU-1, Hep-G2, MCF-7 and SW480 human cancer cell lines.	[81]
	HepG2 cells	Some novel compounds fabricated through azazerumbone conjugation with 2,4-dihydroxychalcones. These compounds showed anti-proliferative activity against the LU-1, Hep-G2, MCF-7 and SW480 human cancer cell lines.	[64]
	Murine hepatoma cells (Hepa1c1c7)	Increases proteasome activity, p62 and microtubule-associated protein 1 light-chain 3 (LC3)-II.	[85]
Breast	ATCC-HTB-22 cells	The conjugation of azazerumbone and 2,4-dihydroxychalcones use for the preparation of novel target compounds. The anti-proliferative activity of these compounds against the LU-1, Hep-G2, MCF-7 and SW480 human cancer cell lines improves compared to azazerumbone or ZER.	[81]
	MDA-MB-231, MCF-7, SUM159 cells	Exposure of cells to ZER resultes in increased cleavage of Notch2 in each cell line.Notch2 activation by ZER inhibits its proapoptotic and anti-migratory response.	[86]
	kinase κB (IKKβ) and the Nuclear factor κB (NF-κB) component proteins	Inhibits the IKKβ kinase that activates the NF-κB and also binds to the NF-κB complex in the TNF pathway. Blocking both proteins can lead to inhibition of cell proliferating proteins to be downregulated and possibly ultimate induction of apoptosis.	
	Human mammary gland adenocarcinoma (MDA-MB-231) cell line	Suppresses the proliferation of MDA-MB-231 cells.	[87]
	Hs578T and MDA-MB231 cells	IL-1β-induced IL-8 and MMP-3 expression, migration and invasion decrease.	
	MCF-7 and MDAMB-231 human cells	Induces significant expression of DR4. Activation of Bax and Bak and is not cytotoxic.	[88]
	Human mammary adenocarcinoma MDA-MB-231 Cell Line	ZER and ZER-NLC markedly suppressed the proliferation ofMDA-MB-231 cells. They arrested MDA-MB-231 cell cycle at the G2/M phase.	[89]
	SKBR3 breast cancer cells	ZER downregulated the level of CD44 expression in CD44+. The induction of CD44 expression by EGFR ligands, EGF or TGF-α, was significantly decreased by ZER treatment.	[90]
Leukemia	MDA-MB-231Cell Line	Proliferation of MDA-MB significantly suppressed by ZER.	[88]
	CML-K562 cells	Inhibits K562 cell proliferation and colony formation capability.	[91]
	WEHI-3B cells	The growth of leukemia cells inhibits.	[81]
	Human myeloid leukemia (HL60)	Drops off the percentage of HL60 cell viability.	[92]
	T-acute lymphoblastic leukemia, CEM-ss cells	Cytotoxic influence on CEM-ss cells and able to apoptosis the T-acute lymphoblastic leukemia.	[93]
	Human T-cell acute lymphoblastic leukemia (Jurkat) cells	Used as a system with sustained-release drug carrier mechanism. ZER activated the caspase-3 and caspase-9 and induced intrinsic apoptotic pathway, cytochrome c release from mitochondria, and PARP cleavage.	[94]
	Human peripheral blood lymphocytes (PBL)	The overall clastogenic effect not significant and is a cytotoxic but not a clastogenic substance in human PBL.	[36]
	Mice thymocytes and splenocytes human PBMC	Proliferation in stimulates time- and dose-dependent manner of human PBMC and mice cells upregulates human cytokine immunomodulatory.	[73]
	Human peripheral blood lymphocytes	At high concentrations induces an apparent substantial increase in the micronuclei frequency.	[95]
Colon	Cells of mice thymocytes, mice splenocytes and human human peripheral blood mononuclear	ZEr activated the mice thymocytes, splenocytes and PBMC with dosage dependent manne.	[96]
	Colorectal cancer cells, (CRC) cells	ZER enhanced radiation-induced cell cycle arrest (G2/M), increased radiation-induced apoptosis and enhanced radiation-induced DNA damage.	[97]
Prostate	Hormone refractory prostate cancer (HRPC) cell lines	Induces antiproliferative and apoptotic influence on PC-3 and DU-145, 2 human hormonerefractory prostate cancer (HRPC) cell lines.	[81]
Brain	Human meningioma cell lines (IOMM-Lee, CH157MN)	Induces apoptosis with enhanced phosphorylation of glycogen synthase kinase 3 β (GSK3β) via inhibition of the Wnt5/β-catenin pathway.	
	Human brain malignant glioma (GBM8401)	GBM8401 cells death induction with a dose-dependent pattern.	[98]
	Human brain malignant glioma (U87MG)	Transfection of GBM 8401 cells with WT IKKα inhibite ZER-induced apoptosis, and ZER markedly decreases IKKα phosphorylation levels with a time-dependent pattern.	[99]
Kidney	Human RCC cell line 786-O	Suppresses STAT3 activation with a dose- and time-dependent pattern in RCC cells.	[100]
	Normal African green monkey kidney cells	Nonsignificant cytotoxicity with 30 µM IC50.	[74]
Ovarian	Normal Chinese hamster ovary cells (CHO)	Has genotoxic produces and cytotoxic influences in high concentrations.	[74]
Miscellaneous	human umbilical vein endothelial cells (HUVECs)	Inhibits HUVECs proliferation, migration and tubule formation.	[101]
	Human oral cancer (KB)	ZER derivatives induces strong cytotoxicity.	[83]
Gastric	Umbilical vein endothelial cells (HUVECs)	Proliferation of cell, VEGF expression and NF-κB activity in AGS cells inhibited by ZER. Reduction in both VEGF expression and NF-κB activity in AGS cells.	[21]
	Human gastric adenocarcinoma (AGS)	Inhibits tumor angiogenesis via reduction of VEGF production and NF-κB activity.	[71]
Skin	Murine epidermal cells (JB6 Cl41)	Murine epidermal cells (JB6 Cl41) Induces heme oxygenase-1 expression by activation of Nrf2.	[83]

**Table 2:** In vivo biological effects of ZER.

Organ	Animal Model	ZER Route	Biological Effects of ZER	References
Lung	BALB/c female mice	Intraperitoneal injection	Effectively controls the growth of tumor and metastasis via delayed progression cancer cell cycle and apoptosis.	[39]
Kidney	Adult male Sprague Dawley rats	Injected intra articularly	Significantly induction in cytosolic glutathione-S-transferase enzyme activity.	[87]
	Six-week-old athymic nu/nu female mice	Injection	STAT3 activation is inhibited in tissues of tumor and the human RCC xenograft tumors growth.	[101]
Leukemia	Male BALB/c mice	Intraperitoneal injection	The growth of leukemia cells inhibits.	[97]
	Chinese Hamster Ovary (CHO) cells and rat bone marrow polychromatic erythrocytes (PCEs)	Intraperitoneal injection	The leukemia cells number in the spleen of BALB/c leukemia mice markedly decreases after 28 days of orally treatment with different doses of ZER-NLC. Inhibits cell proliferation and causes cytotoxicity in the rat bone marrow.	[83]
Miscellaneous	28-Days-old C57BL/6 male mice	Intraperitoneal injection	Significant decreases in content of vascularization and hemoglobin in the plugs from ZER-treated mice, than control mice.	[71]
	Syrian golden hamsters	Oral dose	Decreases hepatic mRNA levels of sterol regulatory element-binding protein-1c and its lipogenic target genes, included fatty acid synthase, acetyl-CoA carboxylase 1, and stearoyl-CoA desaturase 1.	[102]
	Male Sprague Dawley rats	Intraperitoneal injection	Increase with dose-dependent manner in MN production. No significant effect on human PBL by the overall clastogenic.	[97]
	Male Wistar rats	Oral dose	Decreases infiltration of macrophages, IL-1, IL-6, and TNF-α produced by p38 mitogen-activated protein kinase activation.	[103]
Breast	Female severe combined immune deficient (SCID) mouse	Intraperitoneal injection	Retards growth of orthotopic MDA-MB-231 xenografts in association with induction in apoptosis and suppression of cell proliferation (Ki-67 expression).	[90]
	4T1 challenged mice	Oral feeding	ZER controlled the growth of tumor and metastasis by delaying the cancer cell cycle progression and apoptosis.	[39]
Skin	Female HR-1 hairless mice	Topical application	increases of Nrf2 nuclear translocation followed by the promoter activity of HO-1, and also enhances Nrf2 direct binding to the antioxidant response element.	[104]
Liver	Male Sprague Dawley rats	Oral dose	Upregulates heat shock protein expressions in the liver Confers thermoresistant phenotype.	[85]
	Male golden Syrian Hamsters	Oral dose	Improves dyslipidemia by modulating the genes expression involved in the lipolytic and lipogenic pathways of lipids metabolism Decreases hepatic mRNA levels of fatty acid synthase, malic enzyme, sterol-regulatory element binding protein, and 3-hydroxy-3-methyl-glutaryl-CoA reductase.	[21]
Colon	Pathogen-free male Sprague–Dawley rats	Intraperitoneal injection	Lowers expression of PCNA is observed in the rat liver Increases Bax and decreases Bcl-2 protein expression in the liver.	[105]
Paw	Mice	Intraperitoneal injection	Significantly inhibited the production of paw edema induced by carrageenan in dose-dependent.	[102]
Eye	Female imprinting control region (ICR) mice	Oral dose	Inhibits the expressions of NF-κB, iNOS, and TNF-α. Abrogates nuclear translocation of NF-κB.	[106]
